# Supplementary material for: Drop impact printing
Source: Nat Commun. 2020 Aug 28;11:4327. doi: 10.1038/s41467-020-18103-6 (PMC7455714; doi:10.1038/s41467-020-18103-6)
Supplement: Supplementary file 1 — Supplementary Information [file 41467_2020_18103_MOESM1_ESM.pdf]

Supplementary Information

Drop Impact Printing

Chandantaru Dey Modak[1], Arvind Kumar[1,2], Abinash Tripathy[1,3] and Prosenjit Sen[1,*]

[1]Centre for Nano Science and Engineering, Indian Institute of Science, Bangalore, India, 560012

[2]Present Affiliation - Australian Institute of Bioengineering and Nanotechnology, The University of Queensland, Brisbane, Australia, 4072.

[3]Present Affiliation - Department of Mechanical and Process Engineering, ETH Zurich, Sonneggstrasse 3, CH-8092 Zurich, Switzerland.

[*] To whom correspondence should be addressed.

Prosenjit Sen
Email: prosenjits@iisc.ac.in

Supplementary Figures

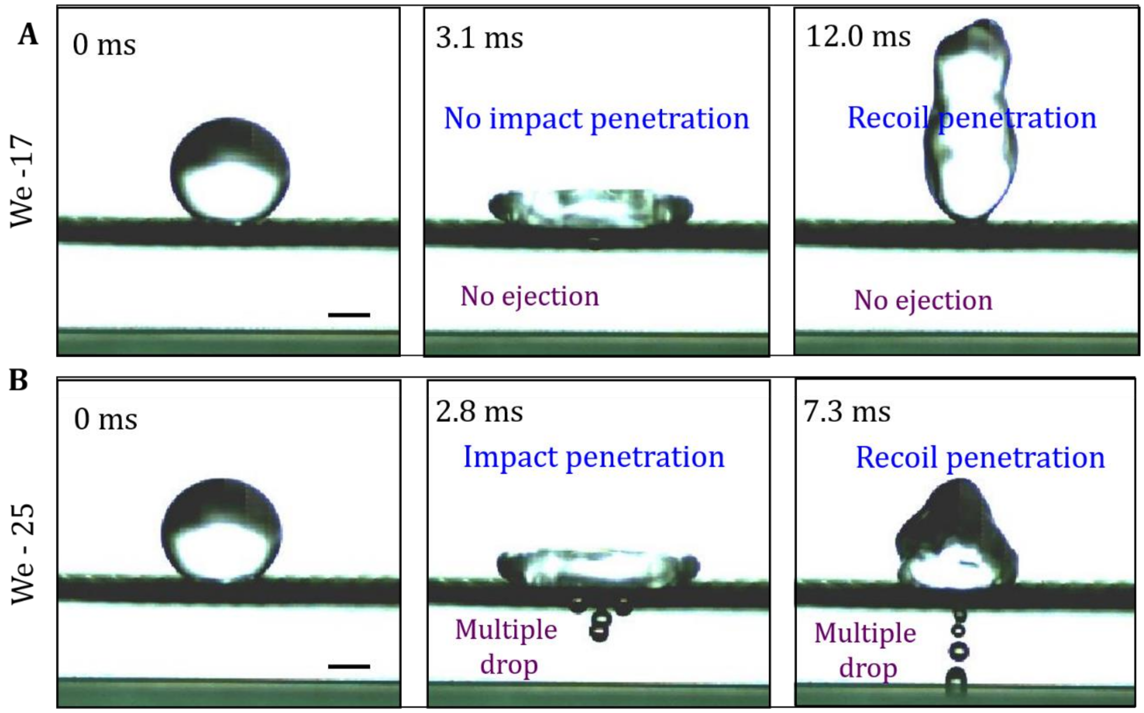

Supplementary Figure 1: Time-lapse images of water droplet when impacting on the superhydrophobic sieve (#0.009) from different heights. (**a**) The drop impact from a height of 2.5 cm (Weber Number (We) - 17), resulting in neither impact penetration nor recoil penetration. **(b)** The drop impact from height of 3.7 cm, resulting in ejection of multiple droplets from single jet. Drop ejection was observed both in impact and recoil penetration, Scale bar – 1 mm.

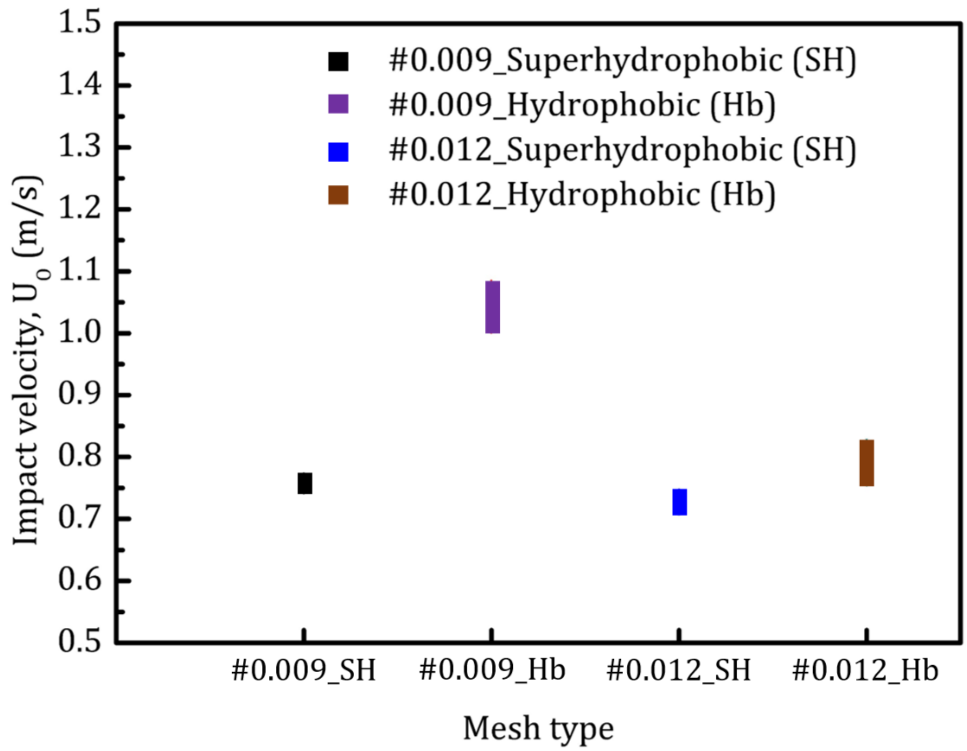

Supplementary Figure 2: The comparison of single drop regime for superhydrophobic and hydrophobic sieve (#0.009 and #0.012). The plot shows the range of impact velocities that ensure single drop ejection when water is used as the printing liquid.

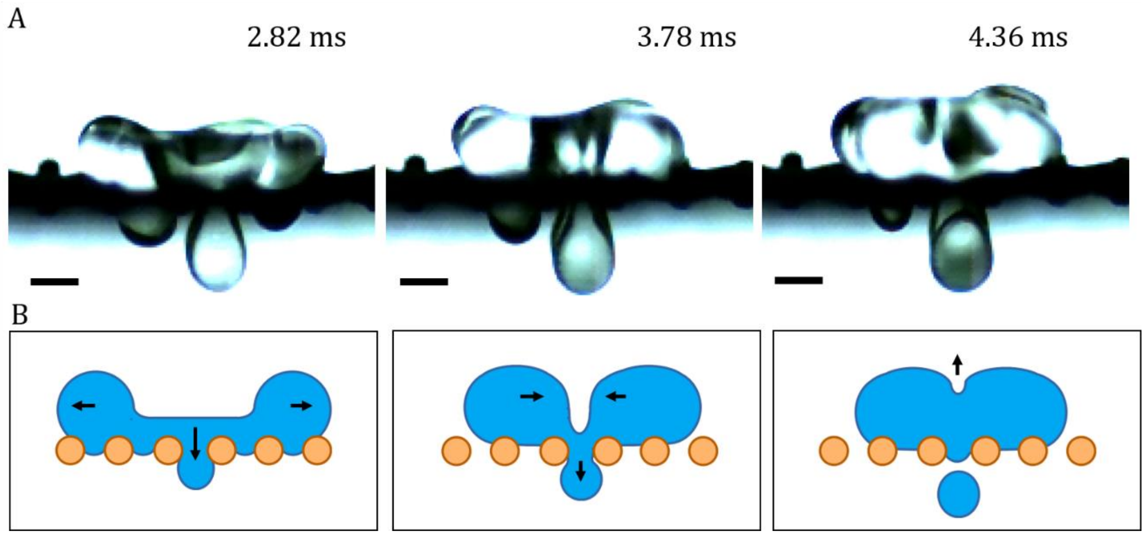

Supplementary Figure 3: Single drop ejection mechanism for sieve type #0.012 is shown using (**a**) time-lapse images (Scale bar – 1 mm) and (**b**) schematic illustration. When drop impacts on sieve, impact jet is formed. As the drop starts to recoil, impact jet is not able to retract back completely and recoil jet pushes the jet further to eject a single drop. In this case, the collapse of top interface of the drop is responsible for single drop ejection.

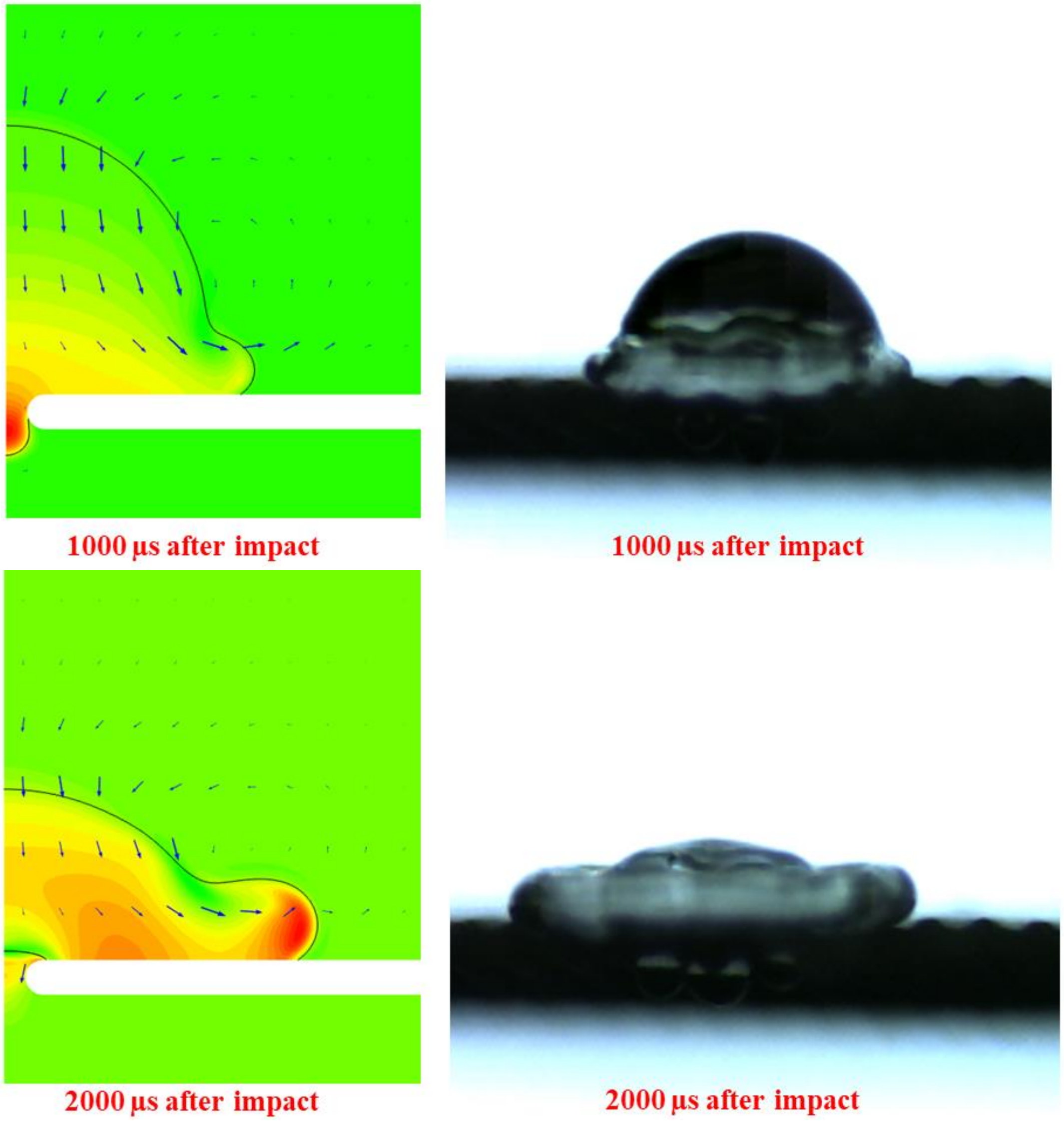
1000 µs after impact
1000 µs after impact
2000 µs after impact
2000 µs after impact

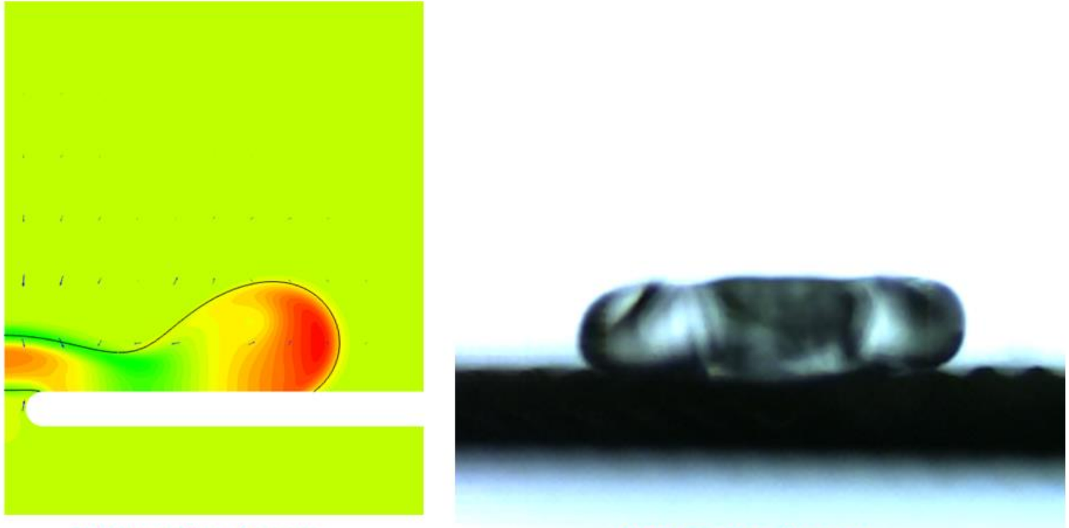

4000 μs after impact   4000 μs after impact

As the simulations do not capture the recoil cavity formation (due to structural difference with the experimental condition), the dynamics start deviating in later stages.

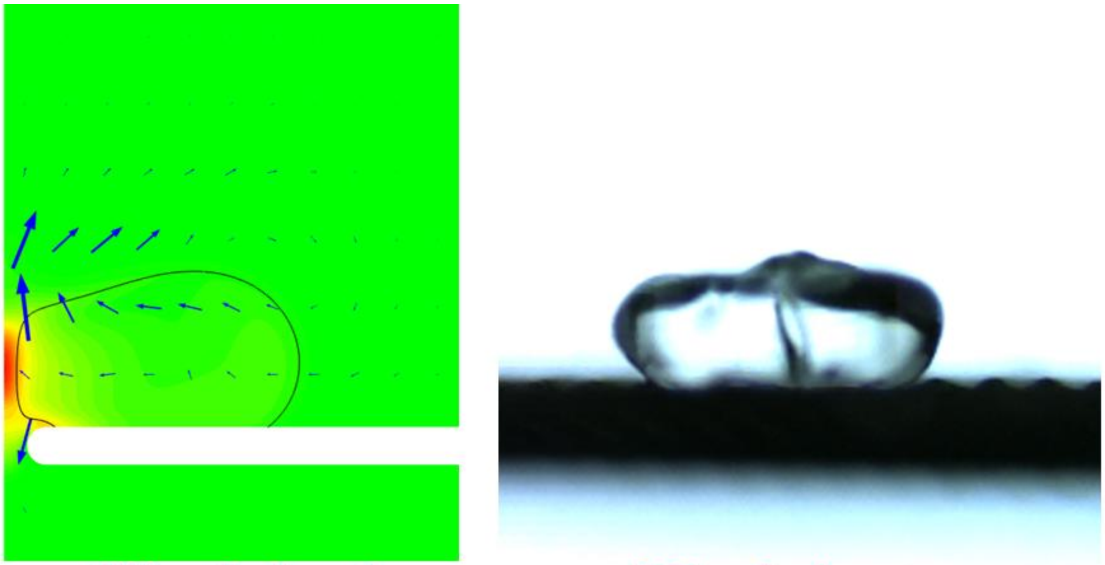

6000 μs after impact   5134 μs after impact

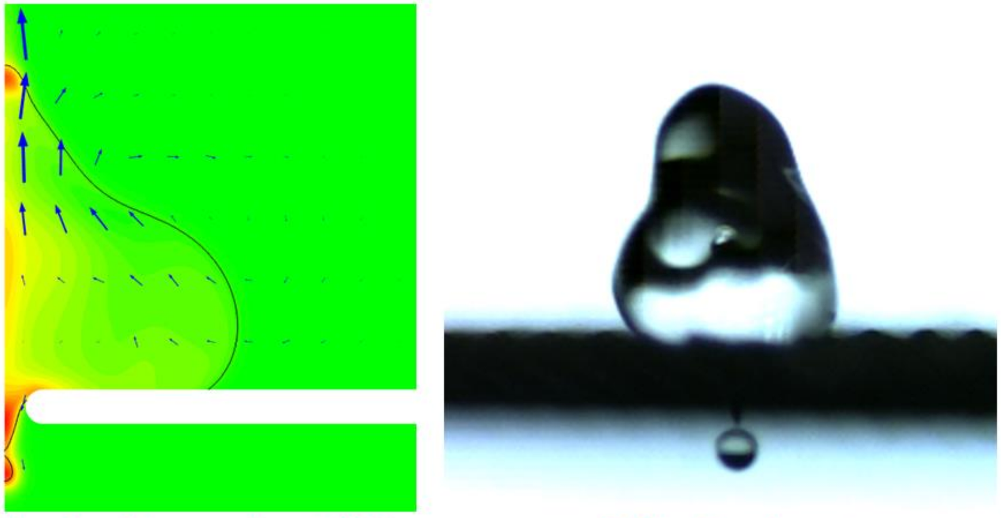

Microdroplet separation at 6860 μs   6634 μs from impact

Despite differences in the simulation and experiments, microdroplets are generated approximately at the same time.

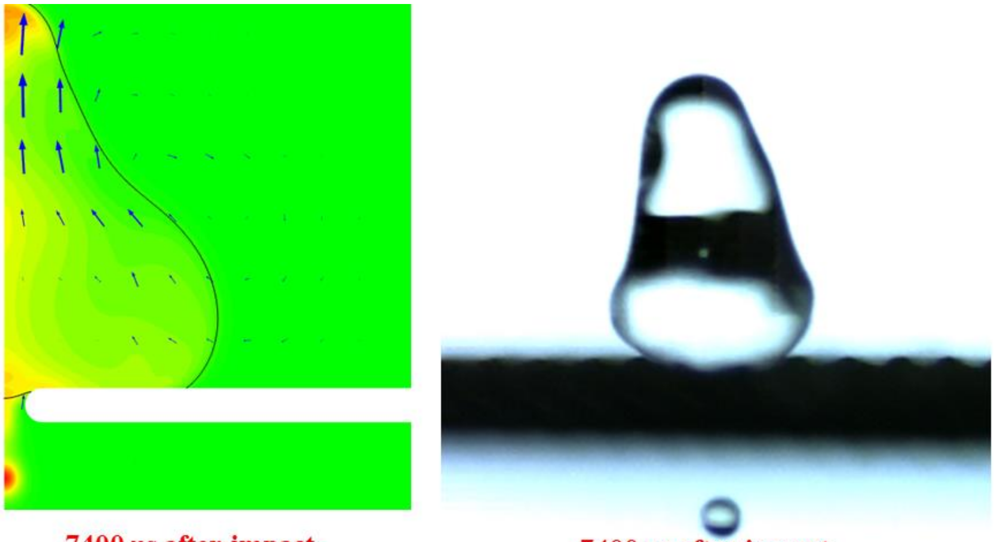

7400 μs after impact   7400 μs after impact

Supplementary Figure 4: Simulation and experimental results at different time scales showing the single drop ejection.

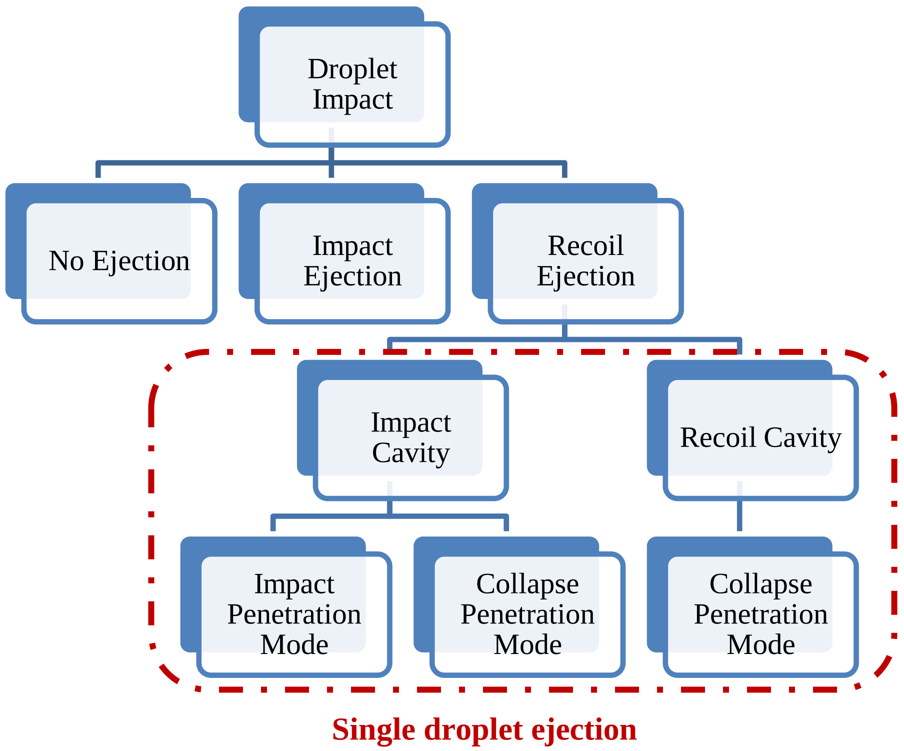

Supplementary Figure 5: Different collapse dynamics resulting from impact of a drop on sieve to generate single drop is shown. The single drop ejection was possible only in recoil ejection. Under recoil ejection, further distinction was made based on cavity collapsed.

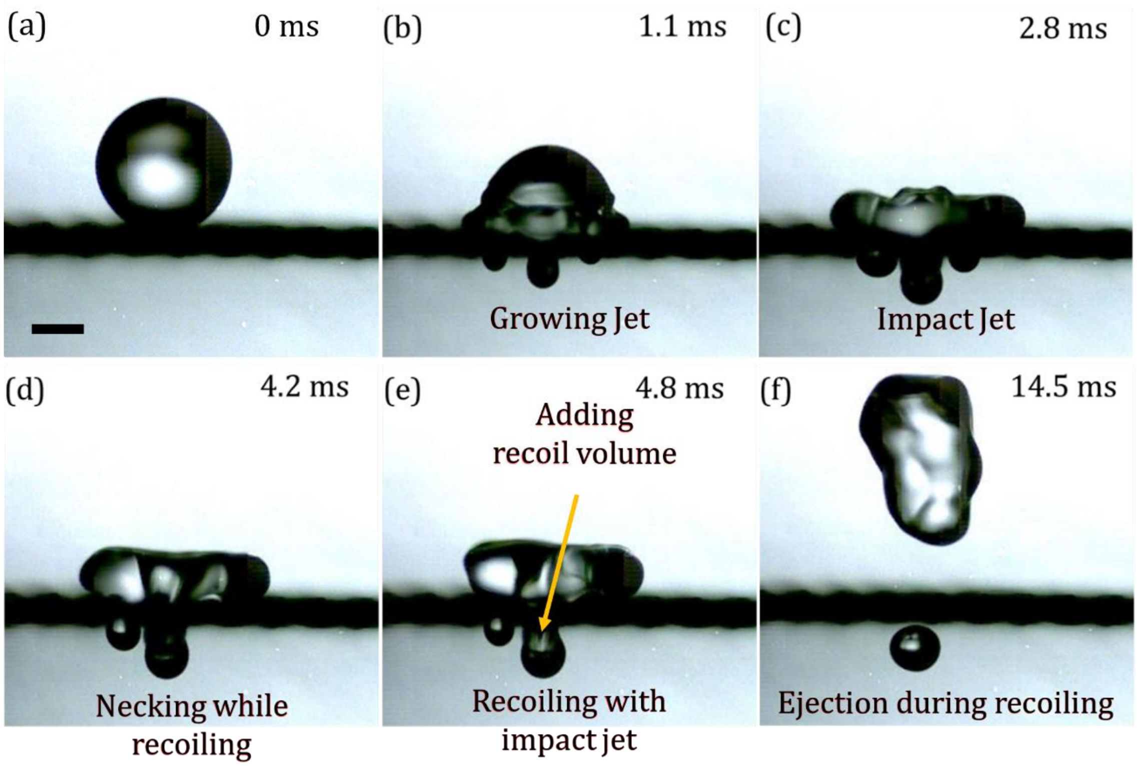

Supplementary Figure 6: Time-lapse images of water droplet impacting on sieve (#0.012). This ejection mode comes under impact penetration mode. In this mode, impact jet contributes to single droplet volume since it is not able to retract back to parent drop completely, Scale bar – 1mm.

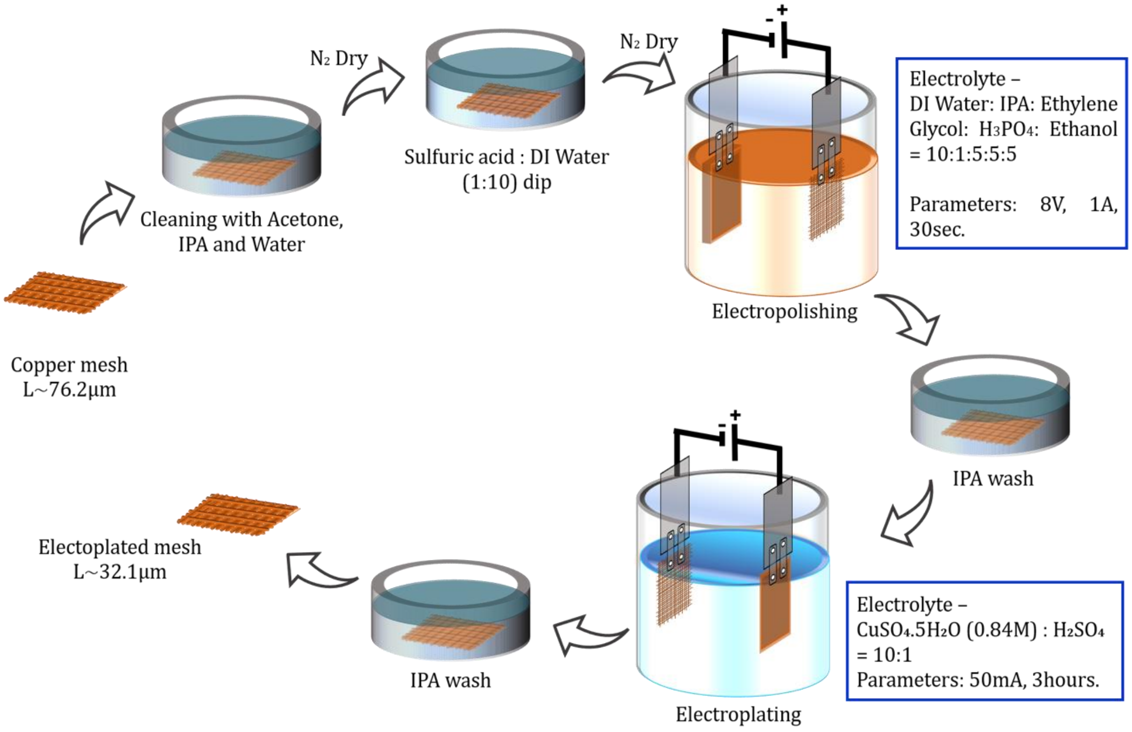

Supplementary Figure 7: Schematic of electroplating process.

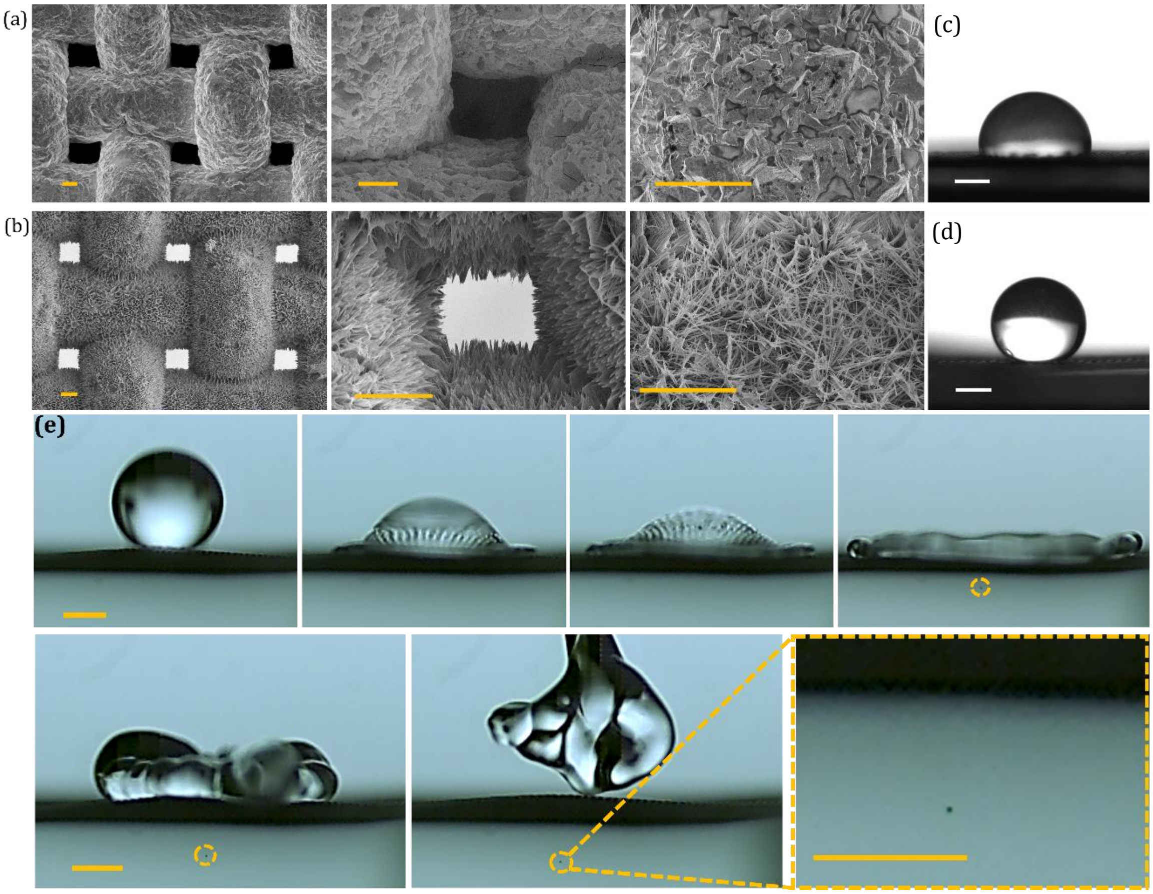

Supplementary Figure 8: Electroplated mesh characterization. (a) SEM images of the electroplated mesh (pore opening - 32.1 μm, wire size - 94.7 μm), Scale bar - 20 μm. (b) SEM images of etched superhydrophobic electroplated mesh (pore opening - 25.2 μm, wire size -101.2 μm), Scale bar - 20 μm. (c,d) Static contact angle on electroplated mesh and superhydrophobic etched electroplated mesh (Scale bar – 1 mm) and (e) Time-lapse photographs showing the ejection of the smallest droplet (42 μm) using superhydrophobic electroplated mesh, Scale bar – 1 mm.

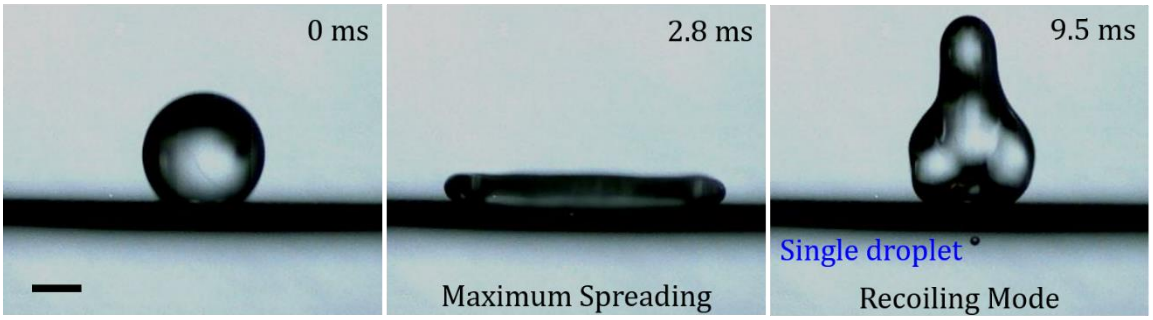

Supplementary Figure 9: Time-lapse photography of viscoelastic drop when impacted on superhydrophobic sieve (#0.0045). The liquid used was 10% (volume percent (v/v)) Xanthum gum-Water solution, Scale bar – 1mm.

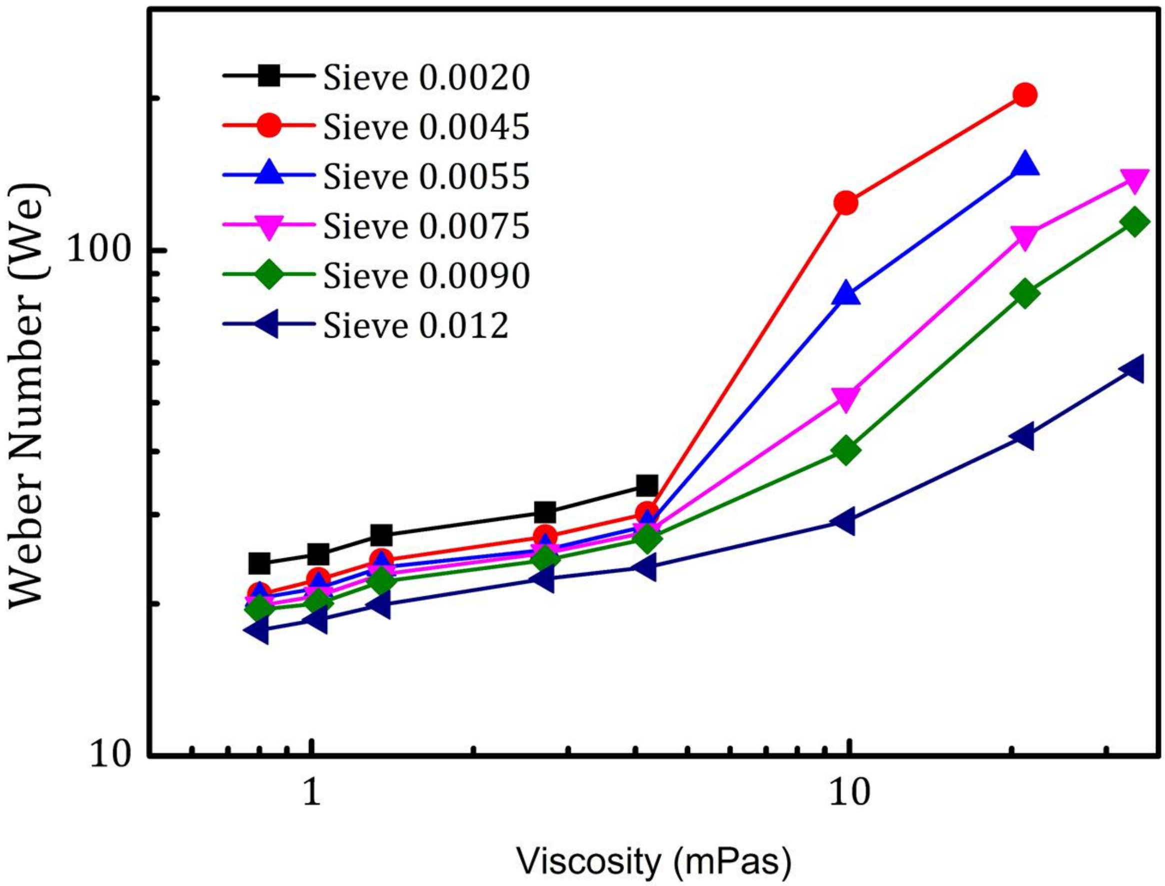

Supplementary Figure 10: Weber number corresponding to single drop ejection with varying viscosity for different sieves are shown. The liquid used is Glycerol-Water mixture of different concentrations.

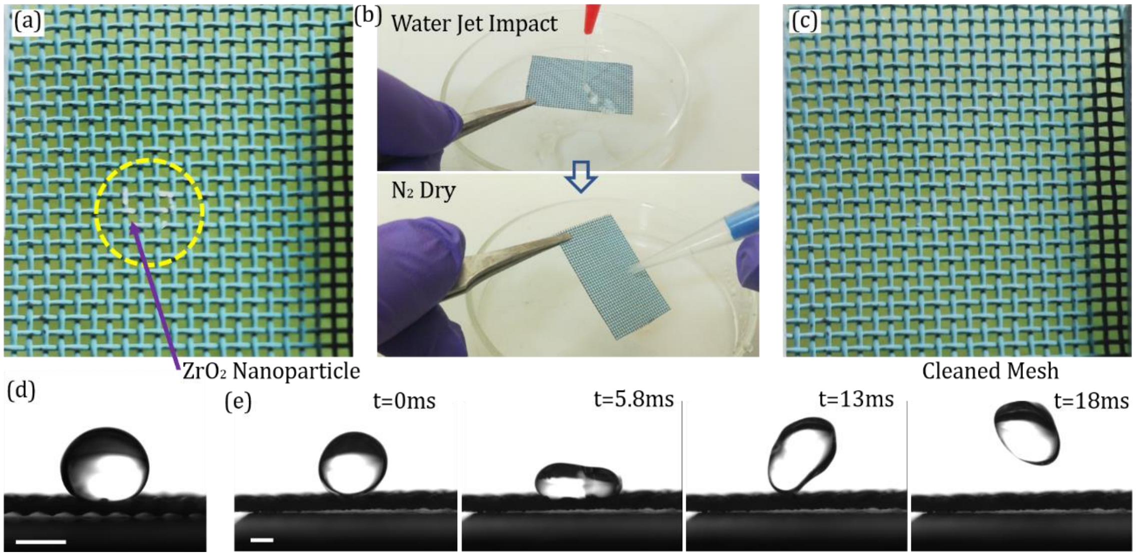

Supplementary Figure 11: Sieve contamination characterization. (a) pinned nanoparticles on SH sieve (#0.012) after 1000 droplets impact. (b) cleaning process of sieve after contamination (water jet impact and N_2 purging). (c) Mesh after cleaning. (d) Static contact angle on cleaned sieve, Scale bar – 1mm and (e) Water droplet repellence test by impacting drops on superhydrophobic cleaned mesh, Scale bar – 1mm.

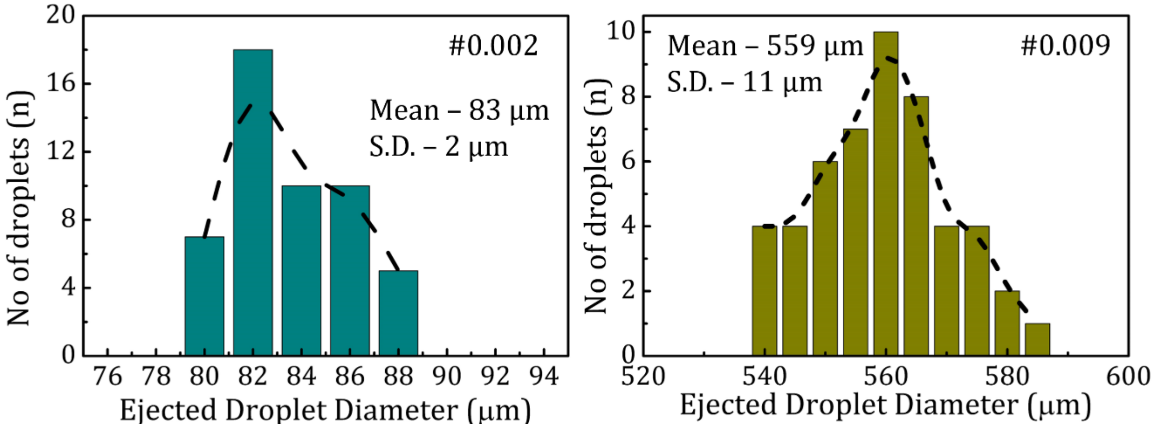

Supplementary Figure 12: The printed feature droplet size distribution has been shown for sieve #0.0020 and #0.009.

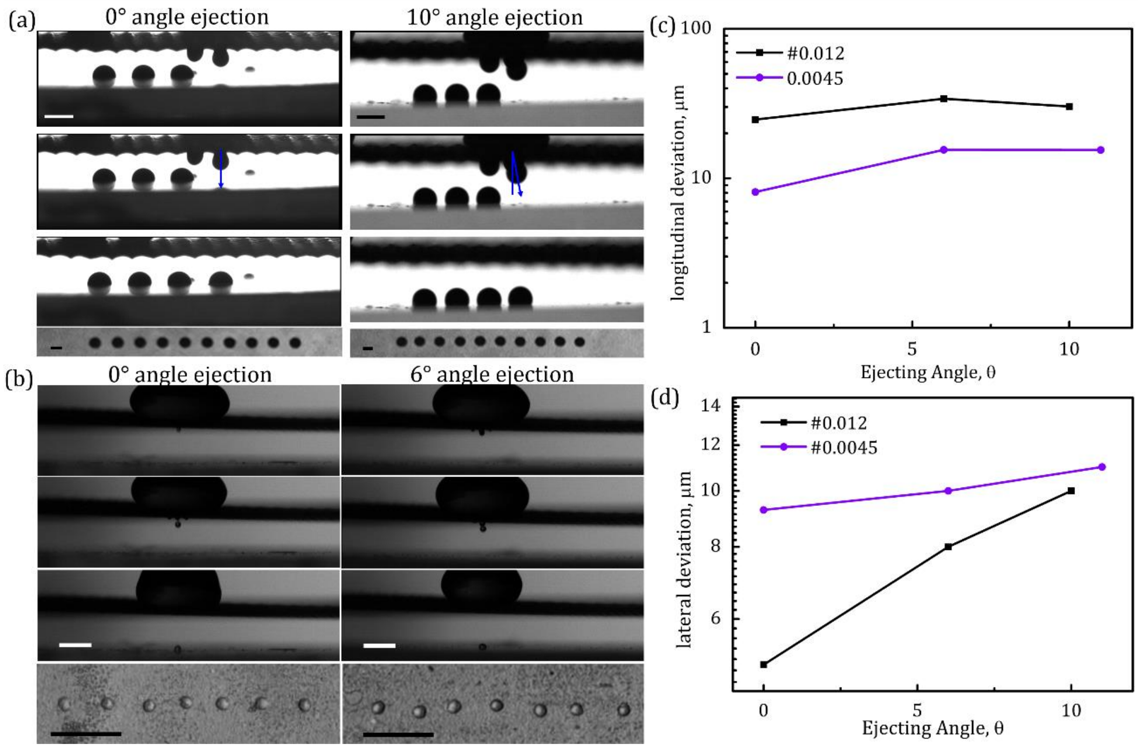

Supplementary Figure 13: Position accuracy calculation: (a) 0° angle ejection and 10° angle of ejection for mesh #0.012 (Scale bar – 1mm) (b) 0° angle of ejection and 6° angle of ejection for mesh #0.0045 (Scale bar – 1mm) and plot between (c) longitudinal deviation (standard deviation) versus ejection angle for mesh #0.012 and #0.0045, (d) lateral deviation (standard deviation) versus ejection angle for mesh #0.012 and #0.0045.

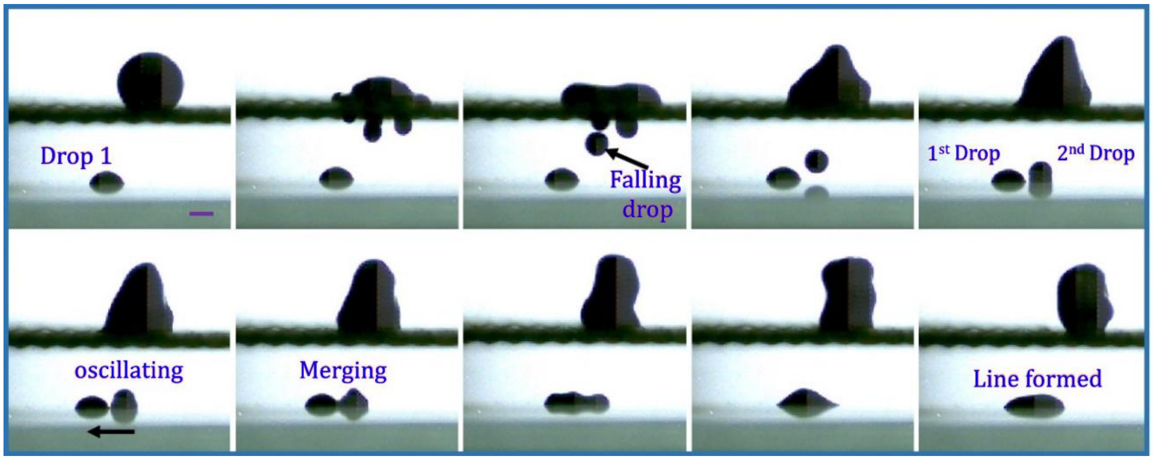

Supplementary Figure 14: The sequence of images showing the ejecting silver ink droplet and merging with the neighborhood drop to form a line. The drop was observed to oscillate and then it merged with neighborhood droplet. The droplets were printed on a glass slide embedded with scotch tape. The spacing between the drops was kept between 150 μm to 200 μm for printing droplet volume of 3 μL, Scale bar – 1mm.

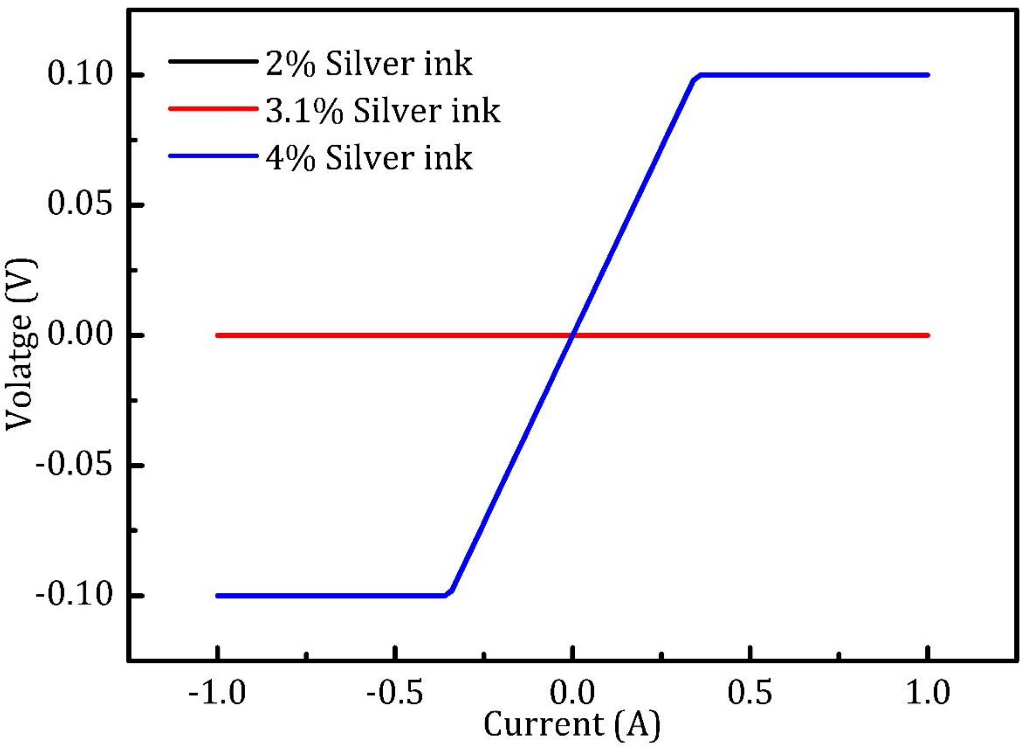

Supplementary Figure 15: Voltage versus current curves for different silver ink concentrations. The optimization of the silver ink line was performed using mesh type #0.009.

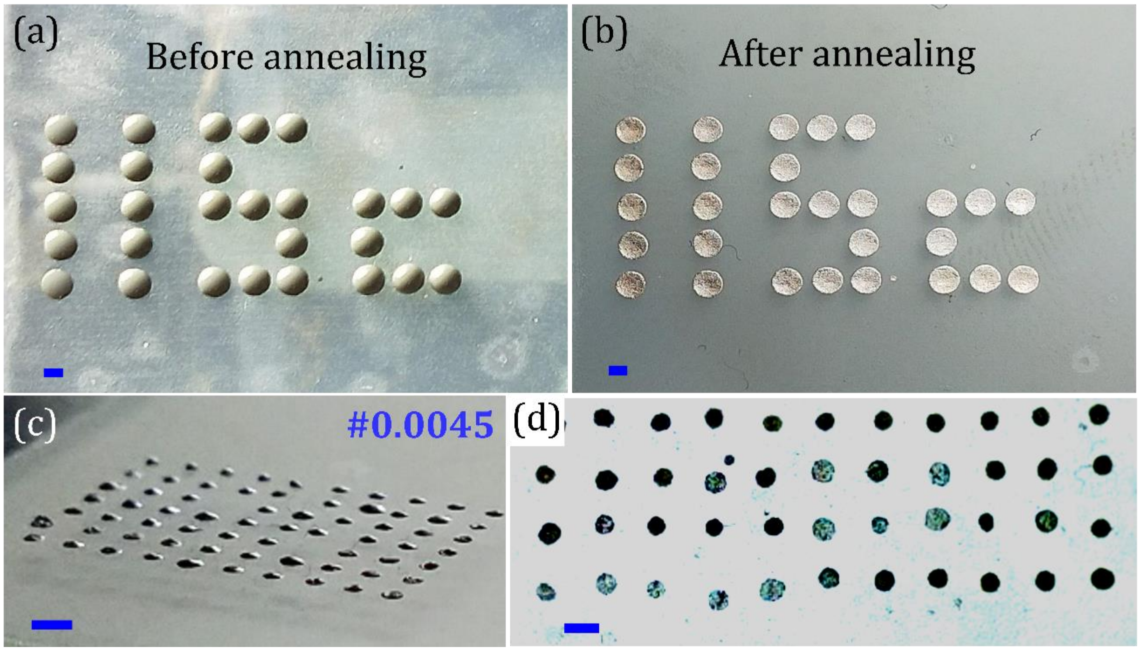

Supplementary Figure 16: Microscopic images of a printed logo of IISc using drop impact printing technique showing patterned silver ink droplet (a) before annealing and (b) after annealing. The printing was carried out with mesh type #0.012 with droplet volume of approximately 0.35 μL. Large area array printing capability of the technique was explored using mesh type #0.0045 showing patterned silver ink droplet from different view, (c) side angled view, and (d) top view. The printed droplet volume was approximately 3 nL. Scale bar – 500 μm.

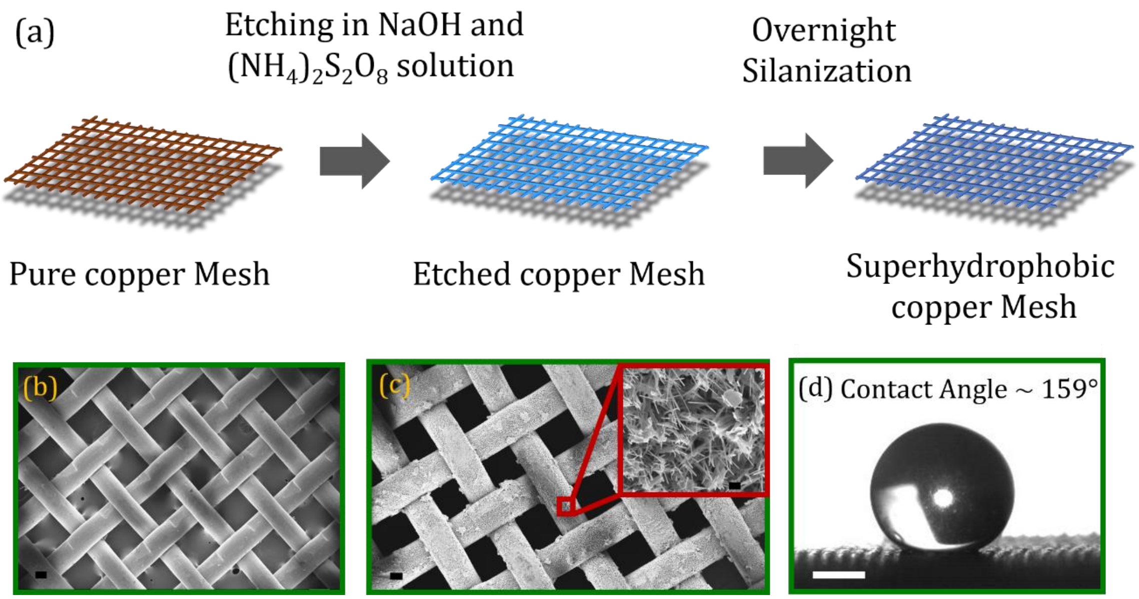

Supplementary Figure 17: (a) Schematic showing etching of clean copper sieve followed by silanization to obtain superhydrophobic sieve. SEM characterization of sieve showing for (b) Clean copper sieve, Scale bar – 100 μm. (c) Etched superhydrophobic sieve (Scale bar – 100 μm) and inset showing the nanowires (Scale bar – 2 μm) that were created over the surface using etching. (d) Contact angle measurement showing the static contact angle on superhydrophobic sieve (~159°), Scale bar – 1mm.

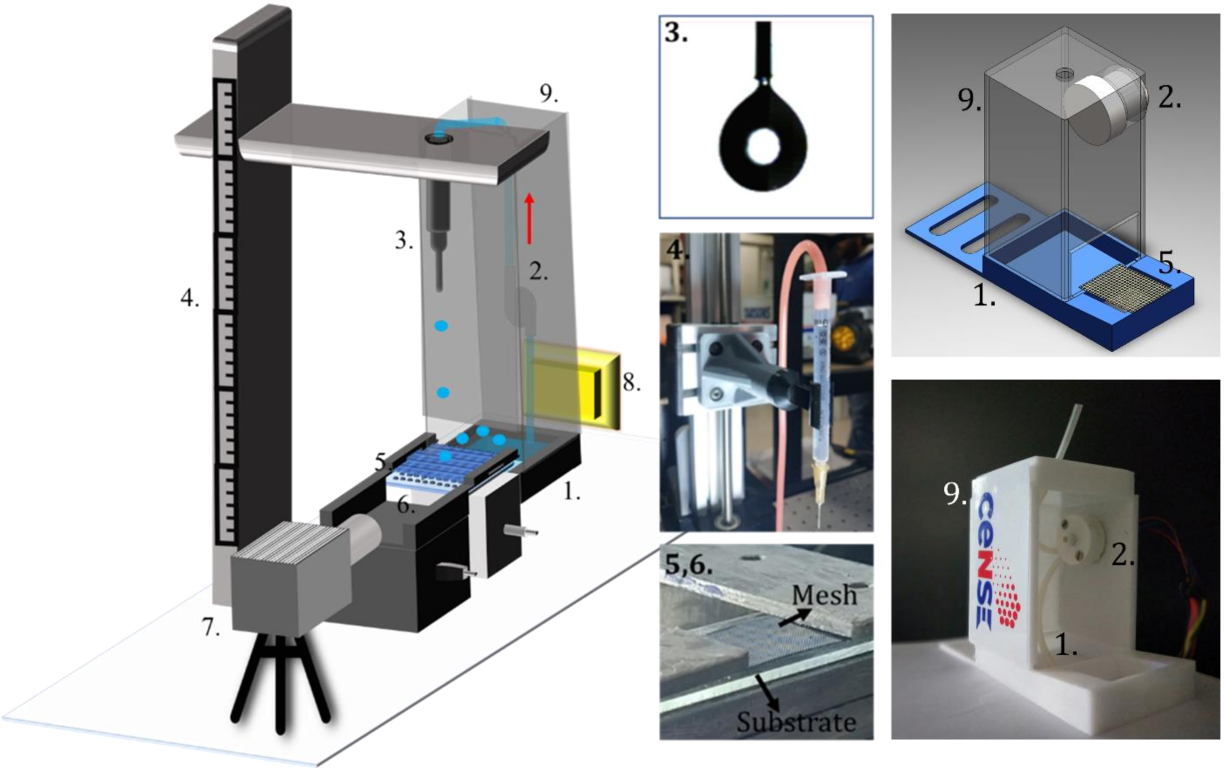

Supplementary Figure 18: (a) Drop impact printing setup with recycling unit.

1. Ink reservoir for recycling of ink
2. Peristaltic pump (pumping ink to the syringe)
3. Mother droplet generation using a syringe
4. Automated z stage for height manipulation
5. Holding platform for mesh
6. Substrate holder fixed with XYZ automatic stage
7. High-speed camera
8. Diffused light
9. Sealed cabinet

Supplementary Tables

Mesh Type [#]	Pore opening, mm (L)	Wire diameter, mm (W)	% Opening
0.012	0.5334	0.3048	40
0.009	0.2794	0.2286	30
0.0075	0.2286	0.1905	30
0.0055	0.1778	0.1397	31
0.0045	0.1397	0.1143	30
0.0020	0.0762	0.0508	35

Supplementary Table 1: Sieve properties.

Mesh Type#	Impact height, cm	Impact Velocity, cms^{-1}	Weber number, We	Reynolds number, Re	Ohnesorge Number, Oh
0.012	2.55	70.718	17.74	2201.4	0.0058585
0.009	2.8	74.104	19.48	2306.8	0.0080947
0.0075	2.85	74.762	19.83	2327.3	0.008949
0.0055	2.95	76.063	20.52	2367.8	0.0101472
0.0045	3.00	76.705	20.87	2387.7	0.0114476
0.0020	3.45	82.257	24.00	2560.5	0.0155002

Supplementary Table 2: Dimensionless numbers of water droplet printing.

Liquid Code	Concentration (v/v)%	Density, kgm^{-3}	Viscosity, mPas	Surface Tension, Nm^{-1}
W	0	997.08	0.8007	0.07025
10GW	10	1020.7	1.03	0.06993
20GW	20	1045.25	1.35	0.06949
40GW	40	1097.1	2.72	0.06835
50GW	50	1123.75	4.21	0.06762
65GW	65	1164.75	9.85	0.06668
75GW	75	1191.95	21.2	0.06535
80GW	80	1205.45	33.9	0.06482
85GW	85	1218.7	58	0.06426

Supplementary Table 3: Fluid properties
A. Newtonian Fluid – Aqueous Water-Glycerol Solution

Liquid Code	Concentration (v/v)%	Density, kgm^{-3}	Viscosity, mPas	Surface Tension, Nm^{-1}
12EW	12.4	888.07	0.8405	0.04753
24EW	24.5	864.40	0.671	0.03797
36EW	36.2	839.97	0.523	0.03298

B. Newtonian Fluid – Ethanol-Water Solution

Liquid Code	Concentration (v/v)%	Density, kgm^{-3}	Viscosity, mPas	Surface Tension, Nm^{-1}
1PEG	1	999.0791	0.67	0.0589
2PEG	2	1000.723	1.2	0.0555
5PEG	5	1005.681	2.9	0.0518
7PEG	7	1009.01	4.12	0.0511
10PEG	10	1014.037	6.1	0.0508

C. Non-Newtonian Fluid – Aqueous PEG Solution

Author Citation	Technique	Surface Tension, mNm^{-1}	Maximum Viscosity, mPas	Ink
Blazdell et. al.[1]	Continuous Inkjet; (BIO.DOT)	-NA-	< 0.01	Ethanol + Dispersant + Polyvinyl butyral, for charging ink (ammonia and acetic acid)
Xiang et. al.[2]	Drop on Demand Piezo Inkjet	21.2	0.00951	Propanol (86.8%) + Ethanol (9.6%) + dispersant
Teng and Edirisinghe[3]	Continuous Inkjet; (BIO.DOT)	~25	0.00164	Ammonium nitrate + Dispersant + Ethanol
Slade and Evans[4]	Thermal Inkjet printer	52	0.003	Water (83.3%) + Polyethyleneglycol (6.7%)
Song et. al.[5]	Continuous Inkjet; (BIO.DOT)	>25	0.0054	Methylated spirit, Poly (vinyl butyral) binder, Dispersant, Plasticizer
Mott et. al.[6]	Piezoelectric IBM inkjet	~22	0.0062	Propanol (85.5%) + Ethanol (9.5%)
Windle[7]	Epson Stylus 500C	~72	0.0036	Polyvinyl alcohol (1.2%) + Water (96.3%)
Seerden et. al.[8]	Piezo inkjet printing	~25	0.0038	Paraffin wax (%) + Dispersant (%) + Organic compounds
Zhao et. al.[9]	Piezo inkjet printing	-NA-	-NA-	Octane (56.89%) + Isopropyl alcohol (14.21%) + Wax (2.84%) + Dispersant (11.85%)
Kosmala et. al.[10]	Drop on Demand Piezoelectric inkjet	-NA-	<0.0033	Water (52.75%) + Pluronic F127 (2.25%)
Lee et. al.[11]	Piezoelectric inkjet	-NA-	0.0017	Water (96%) + Tego Dispersant (2%)
Zhou et. al.[12]	Piezoelectric Epson inkjet	~23.8	0.0013	Cyclohexane and dodecane (1:1)
Nallan et. al.[13]	Custom Drop on Demand Piezoelectric inkjet	-NA-	~ 0.023	Hexane (8%) and α-terpineol (72%)
Salari et. al.[14]	Piezoelectric inkjet (HP 61 cartridge)	26	0.0104	Isopropyl alcohol (40) + α-Terpineol (45)
Lee et. al.[15]	Electrohydrodynamic Printing	NA	NA	Toulene + Dispersant
Park et. al.[16]	Electrohydrodynamic Printing	NA	~0.002	Polyethyleneglycol methyl ether (7.5%) + Water (67.5%)
Lee et. al.[17]	Electrohydrodynamic Printing	48	~0.0173	Ethylene glycol (80%)
Yu et. al.[18]	Electrohydrodynamic Printing	32	0.005	Toulene + Dispersant

Supplementary Table 4: Literature study related to
A. Additive manufacturing for different printing techniques.

**Author** [Citation]	**Technique**	**Surface Tension, mNm^{-1}**	**Maximum Viscosity, mPas**	**Solvent (%)**
A. Negro et. al.[19]	Inkjet printer	~53	0.005	Alginate (0.5% w/v) Polyethylene glycol (3%w/v) + enzymes + Ethylenediaminetetraace-tic acid (EDTA, 0.66 mM)
E. Cheng et. al.[20]	Inkjet printer	~53	0.005	Phosphate buffered saline, BSA, MCF-7 breast cancer cells (1,500,000 cells per mL)
Tao Xu et al.[21]	Thermal Drop on Demand Bioprinter	-NA-	-NA-	Sodium alginate, gluronic acid, phosphate buffered saline, Beta-TC6 cells
L. Gasperini et. al.[22]	Electrohydrodynamic Bioprinter	-NA-	-NA-	Medium, Phosphate-buffered saline (PBS), Trypsin/ Ethylenediaminetetra-acetic acid (EDTA) and the alginate solution

B. Bio-based printing applications for different printing techniques.

**Author** [Citation]	**Technique**	**Surface Tension, Nm^{-1}**	**Maximum Viscosity, Pas**	**Solvent (%)**
Sandler et. al.[23]	Inkjet printing	52	0.0031	Paracetamol, caffeine, and theophylline in propylene glycol-water solution (30:70, v/v%)
Lee et. al.[24]	Piezoelectric Inkjet	35.4	0.00599	Poly(lactic-co-glycolic acid) (100 mg/mL), paclitaxel (PTX) (10 mgmL^{-1}), dimethyl-acetamide (DMAc)
Gu et. al.[25]	Piezoelectric Inkjet	43.5	0.0077	6 wt.% Poly(lactic-co-glycolic acid), 2 wt.% rifampicin (RFP) and 2 wt.% biphasic calcium phosphate (BCP)
Cate et. al.[26]	Inkjet printing	-NA-	-NA-	Printing ink: sodium alginate (3 w%), and calcium Chloride (5 w%) in dematerialized water.<br>Encapsulating material: linseed oil, carrageenan (3w%) dissolved in dematerialized water.

C. Food and Pharmaceutical applications for different printing techniques.

Journal Author Citation	Technique	Nanoparticle Type	Mass loading (%)	$D_{nozzle}/D_{particle}$
Blazdell et. al.[1]	Continuous Inkjet; (BIO.DOT)	Zirconia 5 wt % Yttrium oxide	5.3 (vol%)	-NA-
Xiang et. al.[2]	Drop on Demand Piezo Inkjet	Titanium dioxide	5.3 (vol%)	260
Teng and Edirisinghe[3]	Continuous Inkjet; (BIO.DOT)	Zirconia	2.4 (vol%)	650
Slade and Evans[4]	Thermal Inkjet printer	Zirconia	10 (vol%)	250
Song et. al.[5]	Continuous Inkjet; (BIO.DOT)	Zirconia 5 wt % Yttrium oxide	5 (vol%)	600
Mott et. al.[6]	Piezoelectric IBM inkjet	Zirconia, Carbon	2.5 (vol%)	325
Windle[7]	Epson Stylus 500C	Lead zirconate titanate (PZT)	2.2 (vol%)	375
Seerden et. al.[8]	Piezo inkjet printing	Aluminium oxide + Zirconia	30 (wt%)	187.5
Zhao et. al.[9]	Piezo inkjet printing	Zirconia	14.21 (vol%)	111.11
Kosmala et. al.[10]	Drop on Demand Piezo Inkjet	Silver ink	**45 (wt%)**	2100
Lee et. al.[11]	Piezoelectric inkjet	Zinc oxide	2 (vol%)	252.94
Zhou et. al.[12]	Piezoelectric Epson inkjet	Silver ink	20 (wt%)	5000
Nallan et. al.[13]	Custom Drop on demand Piezoelectric inkjet	Gold	20 (wt%)	24000
Salari et. al.[14]	Piezoelectric inkjet (HP 61 cartridge)	Zirconia 3 mol % Yttrium oxide	15 (wt%)	33.33
Lee et. al.[15]	Electrohydrodynamic Printing	Silver	30 (wt%)	20000
Park et. al.[16]	Electrohydrodynamic Printing	PEDOT:PSS	25 (wt%)	1000
Lee et. al.[17]	Electrohydrodynamic Printing	Silver	20 (wt%)	9000
Yu et. al.[18]	Electrohydrodynamic Printing	Silver	30 (wt%)	60000
Wu et. al.[27]	Electrohydrodynamic Printing	Lead loaded stannic oxide	4.7 (wt%)	200

Supplementary Table 5: Literatures citing mass loading and other parameters of ink used by different printing techniques.

**Liquid Code**	**Average particle size, μm**
Titanium Oxide, TiO_2	0.1
Zirconium Oxide, ZrO_2	.2
Zirconium Oxide, ZrO_2	.5
Halloysite nanoclay	1
Polystyrene Beads	10
Polystyrene Beads	20

Supplementary Table 6: Different nanoparticles and size specifications.

	Drop on demand (DoD) (Piezo based) Printing[28,29]	Continuous (Piezo based) Printing	Electrohydrodynamic Printing[30]	Acoustophoretic Printing[31]	Drop Impact Printing, (DIP)
Resolution, μm	25	20[32]	10	37[33#],130	42
Ink Viscosity, mPas	3 - 35	2- 10[34]	~1000	0.5 - 25,000	<33
Ink Surface tension, mNm^{-1}	44 - 54	20 – 35[34]	NA	~25 – 624	32-72
Position accuracy, μm	66[32]	Low	10	60 - 110	10
Mass loading (%)	<20	~10	~30	NA	71
Max particle size, μm	<0.1	<1	<1.5 [35]	10	20
Nozzle diameter, μm	5	60	5-1000	13 - 140	25 – 533
Droplet detachment mechanism	Pressure waves	Pressure waves	Electrohydrodynamic instability	Acoustic focusing	Cavity collapse induced pressure wave
Energy source	Piezo/thermal driven	Piezo/thermal driven	Voltage driven (Voltage<10kV)	Acoustic radiation pressure	Gravity driven
Working distance, mm	1	5-20[29]	4.5 – 5.5	3.15 – 5.15	1-5
Ink palette	Mostly all inks	Conductive charged inks	Conductive inks, viscous inks	Mostly all inks, high viscous inks	Mostly all inks
Drop volume	1 pL – 8 pL	4 pL -1.76 nL[32]	2 pL[34]_ 135 pL[36]	1.15 nL – 2.145 μL	38 pL – 463 nL
Drop height /drop width (single drop)	~0.004[37]	~0.0014[38]	~0.01[39]	NA	~0.3
Drop on Demand	Yes	No	No	Yes	Yes
Cost (printhead), $	100-1000	100-1000	Low cost	NA	9.4
Nozzle Clogging	Yes	Yes	Can be minimized up to some extent	Less clogging	No

NA - Data is not available, # Resolution of acoustic based drop generation techniques (microfluidics nozzle approach) are higher (~37 μm) and similar resolution can be achieved for acoustophoretic printing by optimizing nozzle diameter and its other parameters.

Supplementary Table 7: Comparison of different techniques with drop impact technique.

Supplementary Note 1: Simulation Results

Drop impact simulation has been performed using the phase-field approach in COMSOL (Supplementary Figure 4). Flow field and phase-field were solved in cylindrical coordinate systems to reduce the computational time. As it is not possible to model the sieve in 2D cylindrical coordinate system, we simplified our simulation by modeling only for the central pore. The pore dimension and the wire size are matched with that of sieve #0.009. We modeled impact of water droplet with radius of 1250 μm with an impact velocity of 0.8 ms^{-1}. Although the simulation was able to capture several aspects of the impact phenomenon, but we did not observe formation of recoil cavity. Though the liquid in the pores was observed to recoil back, the amount of fluid recoiling back was insufficient for formation of recoil cavity. This was because only one pore was simulated. The color scheme shows pressure with red representing higher values and green representing lower values. The arrows show flow direction. The size of the arrows represents the magnitude of local velocities. The images on the right are snapshots from a high-speed video for an impact on sieve #0.009.

Supplementary Note 2: Preparation of Printing Solutions

Glycerol-Water Solution and Ethanol-Water solution

Glycerol and ethanol were purchased from SD Fine Chemicals and distilled water is used for preparing solutions. Different concentrations (v/v %) of glycerol-water and ethanol-water solutions were prepared for the experiment. The glycerol water concentration was varied from 10% to 80% and for the ethanol-water solution, it was from 12% to 36%. The solution viscosity, surface tension, and density are measured using Rheolab QC rheometer from Anton Paar, Density meter: DMA™ 4200 M from Anton Paar, and Tensiometer: K20 from Kruss Scientific respectively. The liquid properties of glycerol-water solutions and ethanol-water solutions are listed in Supplementary Table 3A & 3B.

PEG-Water Solution

Polyethylene glycol 4000 was purchased from Sigma Aldrich. PEG of different weight was mixed in 50 mL of distilled water and stirred, till it completely dispersed. The concentration was varied from 1% to 10% (v/v). The liquid properties like surface tension, density, and viscosities are measured. The liquid properties of PEG-water solutions are listed in Supplementary Table 3C.

Nanoparticle Suspensions

Different nanoparticles of varying size (10 nm to 20 μm) were used in the experiment and are purchased from US Research Nanomaterials, Inc. Nanoparticles of different weights were mixed in 50 mL of 10% (v/v) PEG-water solution and stirred for 30 minutes. To have dispersed solutions, suspensions are further sonicated for 1 hour before the experiments.

For nanoparticle size variation demonstration, the concentration of nanoparticles was fixed around mass loading of 8.88% (w/w). And for different mass loading demonstrations, 200 nm Zirconium dioxide nanoparticles were used and the concentration (mass loading) was varied from 0.88% to 71%. The different nanoparticles size specifications are listed in Supplementary Table 6.

Electronic Inks

For electronic ink printing applications three main inks are used in the present study: PEDOT: PSS, silver ink, and graphite ink. PEDOT: PSS (1.3 wt% dispersion in H_2O), silver ink (30-35 wt%), and graphite ink (20-30%) were purchased from Sigma Aldrich and. PEDOT: PSS and graphite ink was used as it. The aqueous silver ink solutions of varying concentrations (1% to 4%, v/v) were prepared by mixing in a 10% (v/v) PEG-water solution. The ink suspensions were sonicated for 1 hour before the experiments.

Polymeric Solutions

Polyacrylic acid (PAA) was purchased from Sigma Aldrich. Polyacrylic acid of 0.5 gm was mixed in 40 mL of distilled water and then stirred for 1 hour. The prepared viscous mixture was used for printing applications. The viscosity of the polymeric solution was measured to be 1.15 mPas.

Printing solutions of Red blood cell (RBC) and MDA-MB-231 cell

Healthy whole blood (procured from RV Diagnostics Centre, Bangalore, India) of 50 µL was suspended in Phosphate-Buffered Saline (PBS) in the ratio,1:20 (v/v) and then the solution was centrifuged at 2000 rpm for 5 minutes. The supernatant part was removed and then the remaining pellet containing RBCs were resuspended in fresh PBS solution (making the total volume of the solution to 1 mL). Further, different volumes of prepared solutions was mixed to PBS (1mL) to prepare solutions of varying cell concentration. Cell counting was performed using hemocytometer.

MDA-MB-231 cells were maintained in Dulbecco's modified Eagle medium (DMEM, HiMedia) supplemented with 10% fetal bovine serum (FBS, Gibco) and 1% antibiotic-antimycotic solution (Gibco) and incubated in a humid 5% CO_2 incubator at 37°C. The medium was replenished every 24 h. The cells were used at passage 3-4. To split the cells, they were washed with PBS twice and detached by 0.2% trypsin–EDTA, and the cells were counted using a hemocytometer.

Supplementary Note 3: Sieve Contamination

We observed some degree of contamination during the drop impact printing. To examine that, we performed drop impact experiments using ZrO_2 nanoparticles (44.4%, weight percent (w/w)) dispersed in a 1:10 mixture of Ethylene Glycol and water. Drop impact using this solution was carried out for approximately 1000 times at one place using the mesh #0.012 coated with Teflon instead of stearic acid (Please refer Supplementary Table

1 for the geometrical parameters of the mesh). After the experiments, we observed some degree of contamination on the spot where drop impacted the mesh as seen in Supplementary Figure 11a. The contamination was easily removed with a mild jet of deionized (DI) water. After washing the samples were purged in N_2 (Supplementary Figure 11b,c). To verify that the superhydrophobicity of the mesh was retained after the wash, we quantified its wettability through measurement of contact angle and contact angle hysteresis (Supplementary Figure 11d,e). Measured contact angle was 157°±3° and hysteresis was <5° (contact angle hysteresis was calculated from the advancing and receding angle of the droplet at the onset when the droplet starts to slide) satisfying the conditions of superhydrophobicity.

Supplementary Note 4: Description of Supplementary movies

Supplementary movie 1. Single droplet printing during recoil ejection using sieve #0.0045.

Supplementary movie 2. Single droplet printing through impact cavity collapsed penetration mode.

Supplementary movie 3. Single droplet printing through recoil cavity collapsed penetration mode.

Supplementary movie 4. Single droplet printing through impact cavity impact penetration mode.

Supplementary movie 5. Ejected droplets with different diameters for different pore openings.

Supplementary movie 6: Smallest droplet ejection using electroplated superhydrophobic sieve.

Supplementary movie 7. Impacting droplet and moving substrate underneath mesh for single droplet printing.

Supplementary movie 8. 3D micropillar printing using sieve #0.012 (pore opening – 533.2 µm).

Supplementary movie 9. Multiple droplets impacting and ejecting successive single droplets in a row.

Supplementary movie 10. Lab-scale prototype of drop impact printing technique.

Supplementary References

1. Blazdell, P. F., Evans, J. R. G., Edirisinghe, M. J., Shaw, P. & Binstead, M. J. The computer aided manufacture of ceramics using multilayer jet printing. *J. Mater. Sci. Lett.* **14**, 1562–1565 (1995).
2. Xiang, Q. F., Evans, J. R. G., Edirisinghe, M. J. & Blazdell, P. F. Solid freeforming of ceramics using a drop-on-demand jet printer. *Proc. Inst. Mech. Eng. Part B J. Eng. Manuf.* **211**, 211–214 (1997).
3. Teng, W. D. & Edirisinghe, M. J. Development of ceramic inks for direct continuous jet printing. *J. Am. Ceram. Soc.* **81**, 1033–1036 (1998).
4. Slade, C. E. Freeforming ceramics using a thermal jet printer. *J. Mater. Sci. Lett.* **17**, 1669–1671 (1998).
5. Song, J. H., Edirisinghe, M. J. & Evans, J. R. G. Formulation and multilayer jet printing of ceramic inks. *J. Am. Ceram. Soc.* **82**, 3374–3380 (1999).
6. Mott, M., Song, J. & Evans, J. R. G. Microengineering of ceramics by direct ink-jet printing. *J. Am. Ceram. Soc.* **82**, 1653–1658 (1999).
7. Windle, J. & Derby, B. Ink jet printing of PZT aqueous ceramic suspensions. *J. Mater. Sci. Lett.* **18**, 87–90 (1999).
8. Seerden, K. A. M. *et al.* Ink-jet printing of wax-based alumina suspensions. *J. Am. Ceram. Soc.* **84**, 2514–2520 (2001).
9. Zhao, X., Evans, J. R. G., Edirisinghe, M. J. & Song, J. H. Ink-jet printing of ceramic pillar arrays. *J. Mater. Sci.* **37**, 1987–1992 (2002).
10. Kosmala, A., Zhang, Q., Wright, R. & Kirby, P. Development of high concentrated aqueous silver nanofluid and inkjet printing on ceramic substrates. *Mater. Chem. Phys.* **132**, 788–795 (2012).
11. Lee, A., Sudau, K., Ahn, K. H., Lee, S. J. & Willenbacher, N. Optimization of Experimental Parameters to Suppress Nozzle Clogging in Inkjet Printing. *Ind. Eng. Chem. Res.* **51**, 13195–13204 (2012).
12. Zhou, X., Li, W., Wu, M., Tang, S. & Liu, D. Enhanced dispersibility and dispersion stability of dodecylamine-protected silver nanoparticles by dodecanethiol for ink-jet conductive inks. *Appl. Surf. Sci.* **292**, 537–543 (2014).
13. Nallan, H. C., Sadie, J. A., Kitsomboonloha, R., Volkman, S. K. & Subramanian, V. Systematic design of jettable nanoparticle-based inkjet inks: Rheology, acoustics, and jettability. *Langmuir* **30**, 13470–13477 (2014).
14. Salari, F., Badihi Najafabadi, A., Ghatee, M. & Golmohammad, M. Hybrid additive manufacturing of the modified electrolyte-electrode surface of planar solid oxide fuel cells. *Int. J. Appl. Ceram. Technol.* (2020).
15. Lee, D. Y., Hwang, E. S., Yu, T. U., Kim, Y.-J. & Hwang, J. Structuring of micro line conductor using electro-hydrodynamic printing of a silver nanoparticle suspension. *Appl. Phys. A* **82**, 671–674 (2006).
16. Park, J.-U. *et al.* High-resolution electrohydrodynamic jet printing. *Nat. Mater.* **6**, 782–789 (2007).
17. Lee, D.-Y., Shin, Y.-S., Park, S.-E., Yu, T.-U. & Hwang, J. Electrohydrodynamic printing of silver nanoparticles by using a focused nanocolloid jet. *Appl. Phys. Lett.* **90**, 81905 (2007).
18. Yu, J. H., Kim, S. Y. & Hwang, J. Effect of viscosity of silver nanoparticle

suspension on conductive line patterned by electrohydrodynamic jet printing. *Appl. Phys. A* **89**, 157–159 (2007).

19. Negro, A., Cherbuin, T. & Lutolf, M. P. 3D Inkjet Printing of Complex, Cell-Laden Hydrogel Structures. *Sci. Rep.* **8**, 17099 (2018).
20. Cheng, E., Ahmadi, A. & Cheung, K. C. Investigation of the hydrodynamics of suspended cells for reliable inkjet cell printing. in *ASME 2014 12th International Conference on Nanochannels, Microchannels, and Minichannels collocated with the ASME 2014 4th Joint US-European Fluids Engineering Division Summer Meeting* (American Society of Mechanical Engineers Digital Collection, 2014).
21. Xu, T., Kincaid, H., Atala, A. & Yoo, J. J. High-throughput production of single-cell microparticles using an inkjet printing technology. *J. Manuf. Sci. Eng.* **130**, (2008).
22. Gasperini, L., Maniglio, D., Motta, A. & Migliaresi, C. An electrohydrodynamic bioprinter for alginate hydrogels containing living cells. *Tissue Eng. part C Methods* **21**, 123–132 (2015).
23. Sandler, N. *et al.* Inkjet printing of drug substances and use of porous substrates-towards individualized dosing. *J. Pharm. Sci.* **100**, 3386–3395 (2011).
24. Lee, B. K. *et al.* Fabrication of drug-loaded polymer microparticles with arbitrary geometries using a piezoelectric inkjet printing system. *Int. J. Pharm.* **427**, 305–310 (2012).
25. Gu, Y. *et al.* Inkjet printed antibiotic-and calcium-eluting bioresorbable nanocomposite micropatterns for orthopedic implants. *Acta Biomater.* **8**, 424–431 (2012).
26. Pieterse, G. *et al.* Novel encapsulation technology for the preparation of core-shell microparticles. *J. Control. release Off. J. Control. Release Soc.* **148**, e8-9 (2010).
27. Wu, H., Yu, J., Cao, R., Yang, Y. & Tang, Z. Electrohydrodynamic inkjet printing of Pd loaded SnO2 nanofibers on a CMOS micro hotplate for low power H2 detection. *AIP Adv.* **8**, 55307 (2018).
28. Derby, B. Inkjet printing of functional and structural materials: fluid property requirements, feature stability, and resolution. *Annu. Rev. Mater. Res.* **40**, 395–414 (2010).
29. Castrejon-Pita, J. R. *et al.* Future, opportunities and challenges of inkjet technologies. *At. sprays* **23**, (2013).
30. Ball, A. K., Das, R., Roy, S. S., Kisku, D. R. & Murmu, N. C. Experimentation modelling and optimization of electrohydrodynamic inkjet microfabrication approach: a Taguchi regression analysis. *Sādhanā* **44**, 167 (2019).
31. Foresti, D. *et al.* Acoustophoretic printing. *Sci. Adv.* **4**, eaat1659 (2018).
32. Park, J. A. *et al.* Freeform micropatterning of living cells into cell culture medium using direct inkjet printing. *Sci. Rep.* **7**, 1–11 (2017).
33. Demirci, U. & Montesano, G. Single cell epitaxy by acoustic picolitre droplets. *Lab Chip* **7**, 1139–1145 (2007).
34. Magdassi, S. *The chemistry of inkjet inks*. (World Scientific, 2010).
35. Huang, Y. *et al.* Study effects of particle size in metal nanoink for electrohydrodynamic inkjet printing through analysis of droplet impact behaviors. *J. Manuf. Process.* (2020).
36. Guo, L., Duan, Y., Huang, Y. & Yin, Z. Experimental Study of the Influence of Ink

Properties and Process Parameters on Ejection Volume in Electrohydrodynamic Jet Printing. *Micromachines* **9**, 522 (2018).

37. Soltman, D. B. Understanding inkjet printed pattern generation 2011: University of California.
38. Mei, J., Lovell, M., Mickle, M. & Heston, S. Continuous ink-jet printing electronic components using novel conductive inks. in *2004 International Solid Freeform Fabrication Symposium* (2004).
39. Kim, S.-Y. *et al.* High-resolution electrohydrodynamic inkjet printing of stretchable metal oxide semiconductor transistors with high performance. *Nanoscale* **8**, 17113–17121 (2016).
