# Supplementary material for: Drop impact printing
Source: Nat Commun. 2020 Aug 28;11:4327. doi: 10.1038/s41467-020-18103-6 (PMC7455714; doi:10.1038/s41467-020-18103-6)
Supplement: Supplementary file 3 — Description of Additional Supplementary Files [file 41467_2020_18103_MOESM3_ESM.pdf]

Description of Additional Supplementary Files

File name: Supplementary movie 1
Description : Single droplet printing during recoil ejection using sieve #0.0045.

File name: Supplementary movie 2
Description: Single droplet printing through impact cavity collapsed penetration mode.

File name: Supplementary movie 3
Description: Single droplet printing through recoil cavity collapsed penetration mode.

File name: Supplementary movie 4
Description: Single droplet printing through impact cavity impact penetration mode.

File name: Supplementary movie 5
Description: Ejected droplets with different diameters for different pore openings.

File name: Supplementary movie 6
Description: Smallest droplet ejection using electroplated superhydrophobic sieve.

File name: Supplementary movie 7
Description: Impacting droplet and moving substrate underneath mesh for single droplet printing.

File name: Supplementary movie 8
Description: 3D micropillar printing using sieve #0.012 (pore opening – 533.2 µm).

File name: Supplementary movie 9
Description: Multiple droplets impacting and ejecting successive single droplets in a row.

File name: Supplementary movie 10
Description: Lab-scale prototype of drop impact printing technique.
